# Indonesian immigrant nurses' stress, distress, and coping: A qualitative study

**DOI:** 10.1016/j.ijnsa.2025.100466

**Published:** 2025-12-05

**Authors:** Mundakir Mundakir, Ferry Efendi, Hema Malini, Reliani Reliani, Diah Priyantini, Chlara Yunita Prabawati, Rifky Octavia Pradipta

**Affiliations:** a Department of Nursing, Faculty of Health Sciences, Universitas Muhammadiyah Surabaya, Surabaya, Indonesia; b Department of Advance Nursing, Faculty of Nursing, Universitas Airlangga, Surabaya, Indonesia; c Department of Medical Surgical Nursing, Faculty of Nursing, Universitas Andalas, Padang, Indonesia

**Keywords:** Coping, Distress, Immigrant, Indonesian’s nurses, Stress

## Abstract

**Background:**

The experience of nurses migrating abroad to work clinically can have a significant impact on their mental health. Stress factors can include adjusting to a new work system, language challenges, and adaptation.

**Purpose:**

The aim was to analyse the stress, distress, and coping conditions of Indonesian nurses returning home after working abroad.

**Methods:**

This was a qualitative descriptive and interpretative phenomenological approach with 30 nurses who returned home after working abroad, recruited using snowball sampling. Data collection was carried out in Indonesia through semi-structured interviews with the participants. Interviews were conducted in Indonesian and lasted approximately 60 minutes. The data were analysed using reflective thematic analysis.

**Results:**

There were three themes. The first was feeling stressed with the work, consisting of sub-themes feeling a high mental burden, experiences that made one cry, difficulty in adapting, overwork in the field, and feeling alone in a foreign country. The second theme was mental health and its impact on life, with the sub-themes of feeling afraid, feeling traumatised, thinking about losing weight, inability to express feelings, and often being a victim of bullying. The third theme was coping mechanisms while working abroad, consisting of the sub-themes of coping was not good enough, feeling relieved to return to Indonesia, surrendering to the situation, being able to manage oneself and one’s mentality, always having to be ready to learn, relieving stress with entertainment, finding a place to express feelings, and strengthening commitment and planning.

**Conclusions:**

Indonesian nurses working abroad faced a number of complex challenges as migrant nurses.

**What is already known?**

• The mental health of nurses working abroad has challenges that impact stress, distress, and coping skills.
• The problem for Indonesian nurses when working abroad is cultural differences and the adaptation process, which varies from individual to individual.
• Indonesian nurses want to work abroad because they hope to improve their economic benefits and gain additional knowledge.

**What this paper adds**

• Themes about mental health obtained from Indonesian nurses returning home after working abroad included feeling stressed at work, the impact of mental health on nurses' lives, and nurses' coping skills abroad.
• The identified themes could be used for a situation analysis of the state of immigration nurses abroad to provide interventions to improve mental health.

## 1 Introduction

The experience of nurse migration abroad to work clinically is a complex phenomenon and is often associated with various stress factors that can have a significant impact on the mental health of these nurses (Efendi, Hadisuyatmana, et al., 2024; Olanrewaju and Loeb, 2024). These stress factors can include adjusting to a new work system, language challenges, and adapting to a different professional environment (Dousin et al., 2021). Migrant nurses, especially those from Indonesia with a multicultural background that is very different from the destination country, are more at risk of mental health problems compared to native nurses or nurses from countries other than Indonesia (Goudarzian et al., 2024; Ung et al., 2024). This is mainly due to significant differences in practice expectations, patient expectations, and cultural norms that apply in the destination country (Pressley et al., 2022). However, it should be noted that not all nurses will experience the same mental health dynamics (Huang et al., 2020), because this depends greatly on the level and type of stress faced, as well as variations in individual responses to such stress (Efendi, Hadisuyatmana, et al., 2024). Some factors that influence this response include individual characteristics, such as mental resilience and adaptability (Raharto and Noveria, 2020), the social and cultural conditions they face in the destination country (Alameri et al., 2024), specific circumstances related to their migration process (Löyttynen et al., 2023), and the level of acceptance and support they receive in the new country (Kinman, Teoh and Harriss, 2020; Hasan et al., 2021). Factors such as ethnicity, sex, and socioeconomic status of a person are interrelated aspects and have a significant influence on the migration experience (Ung et al., 2024) and also affect their ability to adapt, in interacting both with nurses and other health workers in the destination country (Hassanie et al., 2022; Safiye et al., 2023). Given the complexity of the situation, it is not surprising that many nurses, especially those who are migrating for the first time, experience varying degrees of mental instability (Dousin et al., 2021). This condition can manifest in various forms, ranging from mild anxiety to more serious adjustment problems (Löyttynen, Graneheim and Hörnsten, 2023; Kirkbride et al., 2024).

Indonesian nurses have become one of the most sought-after health categories of workers in various countries. Many nurses have started working abroad to improve their competence and experience, and many also hope to change their prospects (Putri et al., 2021; Efendi, Has, et al., 2024). Of the number of Indonesian nurses working abroad, 30 % of the total are in Japan (Efendi, Has, et al., 2024), followed by 25 % working in the Middle East, such as Saudi Arabia, Kuwait, and the United Arab Emirates (Raharto and Noveria, 2023; Tsujita and Oda, 2023; Ung et al., 2024). Nurses working in Singapore account for 25 % of the total working abroad, 15 % work in the United States (US) and Europe, and the remaining 5 % are spread across various other countries (Raharto and Noveria, 2023). These data show that Indonesian nurses are highly-valued in the international labour market, with the largest concentration in the Asia-Pacific and Middle East regions (Raharto and Noveria, 2023; Efendi, Hadisuyatmana, et al., 2024). The large number of Indonesian nurses recruited by hospital institutions abroad also often gives rise to dynamics of jealousy among nurses, both nurses in that country and nurses from countries other than Indonesia (Jang, Son and Lee, 2022). This is one of the triggers for the emergence of bullying of Indonesian nurses (Efendi et al., 2020; Lu et al., 2022; Tallutondok et al., 2023). Nurses working abroad face various challenges that can affect their mental health. Many nurses have experienced symptoms of depression, reaching 35–40 %, especially in the first 6 months of working in the destination country (Li et al., 2022; Ślusarska et al., 2022). As many as 45 % of migrating nurses have reported experiencing significant anxiety disorders, and almost 80 % of migrant nurses experience high levels of stress related to adapting to new cultures and work environments (Safiye et al., 2023; Efendi, Hadisuyatmana, et al., 2024). Nurses who experience burnout syndrome reach 42 % because they experience extreme work fatigue, and many even experience sleep disorders, such as insomnia or poor sleep quality (Yin et al., 2022; Alameri et al., 2024; Sharif et al., 2024). Factors that contribute to mental health problems for nurses include language and cultural differences, especially when nurses have difficulty communicating, work long hours (average 12–14 h per day, unlike Indonesia's 8 h), and feel loneliness, social isolation, and homesickness. Some have reported high work pressure and discrimination in the workplace, especially unfair treatment (Maglalang et al., 2021; Konlan, Lee and Damiran, 2023; Hua et al., 2024).

Although many Indonesian nurses have worked abroad, there have not been many studies exploring their mental health conditions (Efendi et al., 2021). Nurses who worked abroad and returned to Indonesia felt they had earned enough financially or had negative experiences, with many even experiencing trauma. (Lee et al., 2023). We focused on Indonesian nurses who returned from working abroad and explored the experiences of stress and pressure associated with being an immigrant nurse and how these nurses coped with their difficulties (Lu et al., 2022). Indonesia is a developing country with a large number of nurses, which makes it difficult for many of them to find employment domestically. Even when they do secure jobs, their salaries are often too low to support themselves and their families (Raharto and Noveria, 2023; Ung et al., 2024). Migration is driven by the desire of individual nurses to try to improve their lives in the hope of improving their family's economic situation, because many assume that working abroad will yield a higher salary (Efendi et al., 2020; Sharif et al., 2024). More and more Indonesian nurses are working abroad; as a result, many employment agencies and institutions are starting to focus on recruiting nurses to work abroad (Raharto and Noveria, 2020, 2023; Pradipta et al., 2023). However, these immigrant nurses are sometimes considered at risk of violence, isolation, bullying, and exploitation. Reports of poor working conditions, full of stress and bullying from other health workers, are not uncommon (Efendi et al., 2021; Efendi, Hadisuyatmana, et al., 2024; Ung et al., 2024). Furthermore, >40 % of Indonesian migrant nurses have reported high levels of discrimination in the workplace (Raharto and Noveria, 2020; Pradipta et al., 2023). These factors have also been associated with poorer mental health, but the mental health of Indonesian nurses working abroad is rarely discussed in the literature (Raharto and Noveria, 2020). Some researchers have suggested that discrimination is often carried out by senior nurses, particularly against junior nurses from Indonesia, so that they experience discomfort in their work (Hassanie et al., 2022; Raharto and Noveria, 2023). In addition, financial stressors, relationship adjustments, and social isolation have been identified as important factors in influencing the mental health of Indonesian nurses working abroad (Pradipta et al., 2023). Some nurses may not tell others about their condition and make themselves even more depressed; furthermore, nurses have required consultation with a psychiatrist due to mental health conditions (Safiye et al., 2023; Efendi, Hadisuyatmana, et al., 2024). These conditions provide a basis for further studies to analyse the mental health conditions of nurses after returning from migration.

In this study, we used thematic qualitative analysis of interviews conducted with migrant nurses who have previously worked in different countries. Our main objective was to describe and analyse the impact of migration experiences on mental health. We considered the experiences of migrant nurses as motivations for departure, adaptations during their stay in another country, and resilience after migration. The purpose of this study was to analyse the stress, distress, and coping conditions of Indonesian nurses after working abroad.

## 2 Methods

### 2.1 Study design

We used a qualitative descriptive and interpretative phenomenological approach in post-migration nurses. Post-migration nurses were nurses who had returned from overseas migration and did not return to work there. Phenomenology is concerned with how a person perceives their surroundings (Williams, 2021; Scott, 2022). The research benefited from a phenomenological design since it captured the development of themes during data collection, using protocols created for data gathering (Williams, 2021). We carried out this study in Indonesia between October and November 2024.

### 2.2 **Participant**s

Participants in this study were post-migration nurses. A total of 30 nurses were recruited with the criteria of nurses with experience working abroad for at least 6 months, nurses who worked abroad and returned to Indonesia, post-migration nurses with a return of >3 months (Efendi et al., 2020), and willing to participate. All participants had been informed about the study, and all had filled out an informed consent form as a voluntary consent to participate. The authors ended with 30 participants because, during the interview with the last participant, the authors had achieved data saturation.

### 2.3 Procedure

Recruitment was carried out through the snowball sampling method, while sampling was conducted after the emergence of major codes and categories and continued until data saturation. Data collection was conducted in Indonesia through semi-structured interviews, as mutually agreed upon by the researcher and participants. Interviews were conducted in Indonesian and lasted approximately 60 min. All were conducted by the interviewers MM, RL, CYP, and DP. Interviews were conducted via Zoom, and the interviews were recorded. Several general questions were asked about nursing, wards, and demographic questions (age, sex, education, country where they worked, length of work, and current jobs). During the interview process, the researcher used data collection tools consisting of interview guidelines, voice recorders, paper, and field notes to record essential information. In-depth interviews used an open-ended semi-structured interview guide to obtain information related to the objectives of this study. Questions given to participants focused on the reintegration process after returning from working abroad, social support, mental health challenges, coping strategies, and cultural considerations experienced by Indonesian nurses who migrated abroad. All participants were given the same questions until data saturation was met. The quotations in this paper were translated into English by CY, DP, ROP, and FE.

### 2.4 Data analysis

Data were analysed using reflective thematic analysis. Thematic analysis is a useful approach often used in qualitative research, particularly in phenomenological studies (Wiltshire and Ronkainen, 2021; Christou, 2022). The data analysis process involves familiarising oneself with the data, generating initial codes, searching for themes, defining and naming themes, and writing a report (Wiltshire and Ronkainen, 2021). The familiarisation phase involves transcribing, reading, and rereading the data. The next stage involves reorganizing the data to make statements about themes. In the third stage, codes are organised into possible themes. In the fourth stage, themes are examined for their relationship to the coded extracts and the full data set, providing a thematic “map” of the study. The fifth stage involves increasing the specificity of each topic and defining and naming themes. Finally, stories are created to communicate the core concepts developed through the data analysis, and data extracts or stories are used to support the findings. During the data analysis process, participants reviewed and confirmed the findings; therefore, they directly contributed to determining the keywords, categories, and themes.

### 2.5 Ethical consideration

This study received official approval from the Human Research Ethics Centre at a leading private university in Indonesia. Before the interview process began, all participants were given a participant information sheet written in Bahasa Indonesia to ensure complete understanding, and their written consent was obtained. Throughout the entire research process, the principles of anonymity and confidentiality were strictly maintained and upheld. To ensure identity protection, each participant was given a pseudonym that was used consistently throughout the research. All research data, including interview recordings and transcripts, were stored securely in a protected location that was accessible only to authorised members of the research team.

## 3 Results

In this section, we present the themes and subthemes that emerged from the results of semi-structured interviews with 30 post-work-abroad nurses about the dynamics of mental health after working abroad as nurses.

Table 1 describes the sociodemographic characteristics of the participants, which shows that the oldest participant was 50 and the youngest was 26; all were mostly female (83.33 %). Participants' work-abroad locations included Saudi Arabia (70.0 %) and Japan (13.3 %), while the remainder (16.7 %) were in Kuwait, Taiwan, Germany, the US, and England. The length of participants' work experience varied from 1 year to 10 years. About a quarter of the nurses had worked for 5 years, and when the nurses returned to Indonesia, 76.7 % of participants continued to work as nurses, and the remaining 23.3 % worked in the self-employed sector, continued their studies, or became housewives.

**Table 1:** Sociodemographic Characteristics of the Participants.

**Participant**	**Age**	**Sex**	**Education**	**Country Working**	**Length of work**	**Current Job**
P1	38	Male	Diploma	Kuwait	6 Years	Self-Employed
P2	49	Female	Ners	Saudi Arabia	5 Years	Nurse
P3	44	Female	Ners	Saudi Arabia	3 Years	Nurse
P4	36	Female	Ners	Saudi Arabia	4 Years	Nurse
P5	33	Female	Ners	Japan	2 Years	Continuing Master’s
P6	41	Female	Ners	Saudi Arabia	7 Years	Housewife
P7	42	Female	Ners	Saudi Arabia	5 Years	Housewife
P8	50	Female	Diploma	Taiwan	10 Years	Nurse
P9	38	Female	Ners	Saudi Arabia	2 Years	Nurse
P10	26	Female	Ners	Saudi Arabia	2 Years	Nurse
P11	27	Female	Ners	Saudi Arabia	2 Years	Nurse
P12	29	Female	Diploma	Saudi Arabia	2 Years	Nurse
P13	28	Female	Ners	Saudi Arabia	2 Years	Nurse
P14	26	Female	Ners	Saudi Arabia	1 Year	Nurse
P15	38	Female	Ners	Saudi Arabia	1 Year	Self-Employed
P16	33	Male	Ners	Saudi Arabia	3 Years	Nurse
P17	32	Female	Ners	Saudi Arabia	3 Years	Nurse
P18	35	Female	Ners	Saudi Arabia	3 Years	Nurse
P19	31	Female	Diploma	Saudi Arabia	5 Years	Nurse
P20	40	Female	Ners	Saudi Arabia	3 Years	Nurse
P21	41	Female	Ners	Saudi Arabia	3 Years	Nurse
P22	43	Female	Ners	Saudi Arabia	5 Years	Nurse
P23	45	Female	Diploma	Saudi Arabia	5 Years	Nurse
P24	36	Female	Ners	Saudi Arabia	5 Years	Nurse
P25	26	Female	Ners	Saudi Arabia	6 Years	Nurse
P26	38	Female	Ners	Germany	10 Years	Nurse
P27	33	Male	Ners	Saudi Arabia	8 Years	Nurse
P28	32	Male	Ners	United States	2 Years	Self-Employed
P29	35	Male	Ners	Japan	1 Year	Nurse
P30	31	Female	Ners	England	5 Years	Housewife

Notes: refers to Diploma: Vocational Nursing; Ners: Bachelor’s degree in nursing.

### 3.1 Theme 1: feeling stressed with work

Feeling stressed with work was a reflection of the condition of nurses who reported high levels of stress related to work demands and adaptation in another country. Excessive workload, strict discipline, and high expectations contributed to the emergence of stress experienced by nurses. The first theme consisted of the sub-themes of feeling a high mental burden, experiences that make one cry, difficulty in adapting, overwork in the field, and feeling alone in a foreign country.

#### 3.1.1 Sub-theme 1.1 feeling a high mental burden

Feelings arose from within the participant because they faced a situation different from that in Indonesia; although nurses face high workloads in Indonesia, different cultural and environmental conditions may moderate their self-management capabilities there. Many participants reported feeling stressed after working abroad. All recalled experiencing stress when they first began working. This isn't unique to nurses—individuals in various circumstances inevitably face stress(Pressley et al., 2022; Safiye et al., 2023; Ung et al., 2024). How one responds to stress depends on one's coping abilities. Those with good stress management skills tend to be more resilient and can even thrive in challenging situations (Ung et al., 2024).

*"When I was abroad, I was really stressed when I had a lot of patients, I feel like this is a mental burden if it continues" (P10)*

*"The mental burden is very high; I really couldn't take it at the beginning" (P1)*

*"The experience of a very high mental burden initially made me want to resign" (P5)*

#### 3.1.2 Sub-theme 1.2 experiences that make one cry

Participants who worked abroad expressed their feelings by crying. Crying was a choice for some participants to pour out the burden they felt, so that it did not get worse. When they cried, participants reported they felt a little better, although when they returned to work, they faced other stressors. Crying was mostly felt by participants who had just started working or nurses who were not comfortable working abroad, so they finished their contracts under duress while working.

*"I can only cry, even before I remember, when I felt very overwhelmed and burdened. I ran to the bathroom, then I cried there" (P2)*

*"I cried in the bathroom. I wanted to go home because I felt very tired" (P25)*

*"My family knows my struggles, when I told my mother over the phone, and I cried" (P23)*

#### 3.1.3 Sub-theme 1.3 difficulty in adapting

Adaptation was the ability of nurses working abroad to adjust to circumstances and manage the pressure that arose. The adaptation process, according to Calista Roy theory, the author of one of the nursing adaptation models, states that a person's adaptation process is influenced by factors from both the individual and the environment. Adaptation also cannot take place quickly; it requires a process that also depends on the abilities of each individual. The thing that made nurses have difficulty adapting was the difference in operational standards in Indonesia and the country where they worked; cultural differences and characteristics of nurses in other countries often made nurses uncomfortable while working.

*"The difficulty of adapting to the differences in clinical practices was highly challenging; there, the physician's advice is different, pharmacological therapy is different, not like in Indonesia"* (P30)

*"When people from different cultural backgrounds interact, cultural differences may arise, and if these differences are not managed well, they can negatively affect mental health." (P9)*

#### 3.1.4 Sub-theme 1.4 overwork in the field

Participants reported that they were given abrupt work orders or were required to do all the work, so they felt tired.

*"There, there were a lot of independent nursing actions; the work of nurses was really overloaded. I was honestly very overwhelmed and tired" (P3)*

*"Maybe we are trapped in feelings of being alone with an extra burden" (P8)*

*" There, you cannot ask them for help, nurses must be independent, must be able to do everything" (P26)*

#### 3.1.5 Sub-theme 1.5 feeling alone in a foreign country

The participants felt that living in a foreign country would be a new experience where they could feel at home and adapt, but some participants reported they could not do it because they felt alone. For most individuals with an introverted nature, it might be more difficult to face all of this, even though in other countries there were many friends who could be considered as new family, but they could not easily accept, get close to, or pour out all their feelings to their new family.

*"I often felt afraid because I felt alone there" (P11)*

*"I also often meditated in the park and ate ice cream while crying because I felt alone there with no one close to me" (P17)*

### 3.2 Theme 2: mental distress and its impact on life

Stress is characterised by a negative response shown by a person, while distress does not always end with a negative response, because some people can manage stress positively, adapt, and respond positively and healthily (Tsigos et al., 2020). Distress can affect the way one thinks, manages feelings, or acts. Researchers have found that distress can affect the way one makes decisions and takes actions about physical or mental health (Kinman, Teoh and Harriss, 2020; Hasan et al., 2021). Theme 2 shows the sub-themes of feeling afraid, feeling traumatised, thinking about losing weight, inability to express feelings, and being a victim of frequent bullying.

#### 3.2.1 Sub-theme 2.1 feeling afraid

Fear is a natural condition experienced by people who work in different environments and conditions; the fear felt also comes from various causes (Adolphs, 2013; Pogoy and Cutamora, 2021; Christman et al., 2022). Most of the fear was experienced because participants felt alone, and, when an unexpected event occurred, for those who were in a country far from family and friends, it was difficult to get social support.

*"I had an experience of a patient dying, when in the operating room, the condition deteriorated, then the patient was admitted to the intensive care unit and died. I was immediately called by the head nurse in the room. There was a small committee meeting, and I felt scared” (P6)*

*“Fear of working there because everything must be based on standard operating procedures” (P7).*

#### 3.2.2 Sub-theme 2.2 feeling traumatised

Psychological trauma is a type of mental dysfunction that occurs as a result of a traumatic event. When trauma leads to post-traumatic stress disorder, dysfunction may involve physical and chemical changes in the brain, which alter a person's response to future stress(Hassanie et al., 2022; Safiye et al., 2023; Lubotsky and Hanson, 2024). The trauma faced by nurses working abroad caused them not to want to return to working abroad, even if they worked in a country different from the previous one (Christman et al., 2022; Hassanie et al., 2022; Efendi, Hadisuyatmana, et al., 2024). For post-migration nurses who have returned home, their assessment of working life abroad was already bad, so most decided not to repeat the experience.

*"I felt traumatised there. I would also think twice if I were offered to work abroad" (P13)*

*"When a patient died, I had to do a direct report; the direct report lasted for 4–5 h. At that time, I could not sleep. I did not want to eat, could not sleep. I was traumatised" (P21)*

#### 3.2.3 Sub-theme 2.3 thinking about losing weight

The mechanism of stress can have an impact not only on psychology but also on physical conditions. According to the theory of psychoneuroimmunology, psychological stress that is accumulated over time can cause physical health conditions to worsen. The impact felt by nurses is experiencing signs and symptoms such as fluctuating blood pressure, diarrhea, and weight loss (Slavich, 2020; Tsigos et al., 2020; Christman et al., 2022).

*“The adaptation process took quite a long time and made my weight drop from 73kg to 53 kg” (P14)*

*“Adaptation was difficult, I felt too lazy to eat, my weight dropped dramatically, an average of 10 kg in a year, I felt very mentally stressed” (P22)*

#### 3.2.4 Sub-theme 2.4 inability to express feelings

The inability to express feelings and emotions was significant, as participants had difficulty communicating their mental state. This was often due to fear of stigma, lack of appropriate emotional vocabulary, or concerns about social and professional consequences. This inability often exacerbated feelings of isolation and hindered the process of seeking effective help.

"*Because I can't tell my parents, I'm afraid they'll think about my fate here and that becomes a mental burden, but now that I'm home, I feel relieved to be able to go home again" (P24)*

*"I feel like I can't share with my parents" (P28)*

#### 3.2.5 Sub-theme 2.5: often being a victim of bullying

The participants often faced various challenges and difficulties in carrying out their professional duties, where they could try hard to adapt and adjust to a new work environment. But some participants reported they faced one of the most serious problems that Indonesian nurses often faced abroad: intimidation in the workplace, which could have a negative impact on their mental well-being and work performance.

*"Just be careful with nurses from other countries and different cultures, there's often bullying, and we're given unreasonable jobs, so our burden will be even higher" (P18)*

*"I feel like I'm being bullied there" (P11)*

### 3.3 Theme 3: coping mechanisms while working abroad

Coping mechanisms while working abroad included various adaptation strategies used by workers to overcome challenges and pressures. These strategies included efforts to maintain mental health and emotional well-being while away from family and familiar surroundings. Participants developed specific ways to manage stress, build new support networks, and adjust to different cultures and work environments. The third theme consisted of the sub-themes of coping not being good enough, feeling relieved to return to Indonesia, surrendering to the situation, being able to manage oneself mentally, always having to be ready to learn, relieving stress with entertainment, finding a place to express feelings, and strengthening commitment and planning.

#### 3.3.1 Sub-theme 3.1 coping is not good enough

Participants' coping strategies were not yet good enough. Participants showed difficulty in implementing effective and sustainable stress management mechanisms, which resulted in symptoms of distress continuing in daily life.

*"Working abroad really affects my mental state. Different cultures make me have to be able to adapt, considering my age is not yet mature, I found it difficult to adapt to this situation" (P12)*

*“At first I found it hard to control myself; many friends said that your coping must be good, don’t give up easily in a foreign country” (P25)*

*“Sometimes, I am able to deal with stress. It was very difficult for me to adapt” (P26)*

#### 3.3.2 Sub-theme 3.2 feeling willing to return to indonesia

Participants were willing to return to Indonesia after going through a long process of consideration and adjustment. The decision to return to the homeland was based on various personal and professional factors, which ultimately led to acceptance and peace of mind in undergoing this transition. The choice to return to Indonesia became a priority because they felt more comfortable living with their family.

*"My father is old, I'm afraid he'll leave me at any time. Finally, I Legowo (accept), yes, I can gather with my family, even though the salary is small in Indonesia, I can still go home, my main priority is my family" (P20)*

*"I decided to go back to Indonesia, it's okay even if the salary is not high. But the work pressure might be less, I want to go home" (P25)*

*“After I experienced all the difficulties, I decided to return to Indonesia and gather with my family” (P3)*

#### 3.3.3 Sub-theme 3.3 resigned to the situation

Resigned to the situation was a common response reported by participants when facing ongoing stress. This feeling of helplessness often arose after repeated efforts to cope with stress did not produce the expected results. This condition was characterised by a decrease in motivation to seek active solutions and a tendency to accept stressful situations without trying to change them.

*"I surrender to God, I have tried, but there is no fortune" (P19)*

*"Here I accept any job, I am already at the stage of accepting and surrendering, I accept any salary, as long as I am healthy" (P1)*

*"I felt fine if I ended up leaving nursing and choosing to go into business" (P30)*

#### 3.3.4 Sub-theme 3.4 able to manage oneself and one's mental state

Ability to manage oneself and one's mental state effectively through comprehensive emotional control strategies, structured and systematic time management, and maintaining a sustainable life balance were mentioned. These strategies included mindful breathing techniques, daily meditation, and journaling practices to process emotions. In terms of time management, the approach taken included setting clear priorities, breaking tasks into smaller parts, and scheduling regular breaks. This ability included a high level of self-awareness to recognise personal limitations, identify early signs of mental fatigue, and take appropriate initiative-taking actions to maintain ongoing mental health. It was also important to regularly evaluate and adjust the strategies used, ensuring their effectiveness in the long term.

*"Our mental health condition is important; we must be able to manage it ourselves" (P23)*

*"We had to be able to be resilient immediately, so we did not get left behind" (P21)*

*" I did not feel stressed; it all depends on ourselves in managing stress" (P15)*

*"Personally, we must adapt. When we are faced with a new environment, we adapt" (P16)*

#### 3.3.5 Sub-theme 3.5 must always be ready to learn

Many nurses had the perception that working abroad required extra learning because of cultural differences and abilities. It takes deep knowledge and skills to be able to adapt to conditions in countries abroad (Maglalang et al., 2021; Pogoy and Cutamora, 2021; Pressley et al., 2022). However, for nurses who had good coping, they assumed that learning was needed not only abroad, but also in their own country or anywhere. This kind of thinking made nurses have positive thoughts; in addition, participants reported that the risk of experiencing mental health problems was also lower as their readiness improved.

*"You have to ask a lot of questions and learn new things if you want to be successful" (P18)*

*"It's different from our country; you have to be willing to learn if you want to be successful in other people's countries" (P12)*

#### 3.3.6 Sub-theme 3.6 relieving stress with entertainment

Relieving stress with various forms of healthy and constructive distractions was a strategy that was often used. Diversionary activities, such as reading books, listening to music, watching movies, or doing creative hobbies, can help divert the mind from the source of stress.

*"There, I felt stressed, tired of work. I relieved stress by eating chocolate, or sometimes I got fresh air by being alone in the park" (P29)*

*"When I'm abroad, for me, friends are family" (P2)*

#### 3.3.7 Sub-theme 3.7 finding a place to express feeling

The importance of expressing feelings to others was a key aspect of managing mental stress. Sharing feelings and experiences with people one trusted helped ease the emotional burden, provide new perspectives, and create a stronger sense of connection. The ability to communicate feelings openly could also help in identifying sources of stress and finding more effective solutions to overcome them.

*"For my strategy, I liked to confide there, call, remind each other, we told stories and strengthened each other, sometimes it made us laugh" (P5)*

*"I told my story to my friend, using a video call. I felt more relieved" (P19)*

*"I didn't want to keep my problem to myself; I always told other people" (P27)*

*“When I faced difficulties, I chose to talk to my friends instead of my parents. I did not want to worry my parents, and sharing with peers who had similar experiences made me feel less alone” (P20)*

#### 3.3.8 Sub-theme 3.8 strengthening commitment and planning

Strengthening commitment and planning was an important aspect in overcoming work stress. Research participants who managed work stress effectively generally had a strong commitment to prioritising their well-being and developing a structured plan to achieve a better work-life balance. Careful planning included setting realistic deadlines, organising tasks systematically, and scheduling adequate rest time.

*"Stress is definitely there, but I strengthen it with my WhatsApp group and family, I feel that commitment and ideals must be maintained" (P19)*

*"Confident in my abilities, must have positive self-confidence and confidence so that work runs smoothly" (P10)*

### 3.4 Theme 4: the unique experiences of indonesian nurses in arabia, east asia, and europe

This section includes specific and various adaptation strategies used by workers to overcome challenges and pressures in each country or region where they worked abroad.

*"In Japan, I learned self-worth, the value of hard work, and how to practice discipline and respect in my professional role. I still admire and strive to apply these lessons today, while also recognising the effort I have put in" (P5)*

*“In Saudi Arabia, the unfair treatment by senior staff from other Asian countries makes me sad. The workload is often excessive—we nurse from Indonesia are always assigned to handle very ill and terminal patients. When patients died, the lead nurses and seniors did not want to be involved with the issue; it became solely our responsibility. Is this really teamwork? I sometimes wondered. We wished we had protection from the nursing organisation here, but nursing organisations in Saudi Arabia and Kuwait operate quite differently” (P14)*

*"England and other European countries prioritise skills and knowledge. Once you have passed the language and skill competency exams, your integrity will be key to proving your true capabilities, and you will be able to work professionally. Sometimes winter blues are truly challenging, but with a warm heart and high spirit to finish your job professionally, your path will become warmer, and your efforts will pay off." (P10, P5)*

## 4 Discussion

We elaborated on reported significant mental health challenges, persistent fear, cultural friction, and severe difficulty adapting to the new environment. Despite differing motivations for working abroad, most participants reported similar experiences: profound concerns about career, life prospects, and intense homesickness, especially for family left behind (Efendi, Hadisuyatmana, et al., 2024), a feeling of loneliness in a foreign country (Kinman, Teoh and Harriss, 2020), and constant worry about the possibility of making mistakes in carrying out their professional duties (Safiye et al., 2023). These pressures are further exacerbated by the demands of maintaining high-performance standards in a foreign work environment (Goudarzian et al., 2024). We have shown that the transnational roles carried out by participants placed significant additional pressure on them. This is mainly due to the growing perception in society that working abroad is a very promising job and can certainly change the economy of life drastically (Pressley et al., 2022). These high expectations from family and society created their own mental problems, where nurses feel burdened with the responsibility for their own adaptation challenges in the destination country (Hasan et al., 2021).

A number of significant causes of mental distress make it difficult for nurses to continue working abroad (Riftana et al., 2024; Tanaka and Yoshimura, 2024). Indonesian nurses working abroad face significant psychological challenges due to their dual roles as health professionals and immigrants (Kinman, Teoh and Harriss, 2020; Safiye et al., 2023). Lee et al. (2023) found that three-quarters of Indonesian immigrant nurses experienced moderate to high levels of stress in their first 2 years abroad. A qualitative study found that the main factors causing stress included language, communication differences, cultural adaptation pressures, financial responsibilities towards family in Indonesia and high professional demands (Widiasih et al., 2021).

A primary concern was the frequent bullying incidents nurses faced, which aligns with a previous study that showed frequent bullying can lead to deep and enduring psychological effects (Peris-Ramos et al., 2024). The bullying experienced primarily took the form of disproportionate workloads, aligning with prior findings of unreasonable schedules, excessive tasks, and unrealistic expectations (Efendi et al., 2020; Raharto and Noveria, 2020). An excessive nursing workload can ultimately affect not only their mental health but also the quality of care they can provide, which relates to Theme 2, mental distress and its impact on life. Studies on bullying behaviour towards nurses in various countries have shown serious impacts on their mental health and professional performance (Putri et al., 2021; Löyttynen, Graneheim and Hörnsten, 2023). Researchers have revealed various aspects of this problem, including its prevalence, impact, and handling strategies. Workplace bullying can reduce nurses' job satisfaction by up to 45 % and increase turnover rates by 30 % (Jang, Son and Lee, 2022). One group of researchers revealed that 67 % of nurses who experienced bullying reported a decrease in the quality of patient care, with a 25 % increased risk of medical errors (Jang, Son and Lee, 2022; Sharif et al., 2024). A longitudinal study showed that hospitals with high levels of bullying experienced a 40 % decrease in patient satisfaction scores, compared to institutions with a more positive work environment (Raharto and Noveria, 2020; Lu et al., 2022). Studies from multiple countries have confirmed that workplace bullying has a systemic impact on the quality of healthcare, staff productivity, and overall organisational well-being (Al-Momani, Al-Ghabeesh and Qattom, 2023; Galanis et al., 2024; Nielsen et al., 2024).

Participants reported exhaustion and diminished hope, consistent with prior findings of mental fatigue, decreased motivation, and difficulty concentrating on important tasks (Mucci et al., 2019). Participants also reported that this often created a negative cycle, similar to the findings of Dousin et al. (2021) that work pressure increased stress levels, which in turn affected work performance. Few previous researchers have explored this theme of work stress in depth. Eriksson et al. (2023) found that 78 % of international workers experience significant and ongoing mental strain, while 45 % had repeated crying episodes related to workplace stress. These findings highlight the complex adaptation difficulties within expatriate workers experienced significant adaptation challenges in their first 6 months, particularly in communication, cultural adjustment, and team dynamics (Huang et al., 2020).

We showed that workload and work pressure were related to stress. Previous researchers have identified that work overload was the main stress factor for 82 % of their participants, with manifestations ranging from physical exhaustion to decreased productivity (Rodriguez‐Arrastia et al., 2021). Meanwhile, 70 % of international workers linked health and performance to their experiences (Eriksson et al., 2023). Nurses often report isolation, and 55 % have reported ongoing fear of professional failure that impairs daily performance (Nielsen et al., 2024; Riftana et al., 2024). The impact of these stressors in a broader context, finding that the combination of work overload and ongoing social isolation increases the risk of burnout by 300 % (Popa et al., 2022; Bao, Liu and Zhang, 2024).

The theme of mental health distress showed that participants experienced fear and trauma that impacted their physical condition, including weight loss and sleep disturbances. Participants also reported an inability to express feelings and were often victims of bullying. Participants who experience mental distress show significant symptoms of fear and trauma, with manifestations ranging from excessive anxiety to persistent feelings of depression (Löyttynen, Graneheim and Hörnsten, 2023). These conditions had a direct impact on nurses' physical health; this aligns with such conditions as substantial weight loss, persistent sleep disturbances, and decreased immune system function (Kinman, Teoh and Harriss, 2020; Hasan et al., 2021). Furthermore, nurses had difficulty expressing their feelings related to mental distress experienced at work; this aligns with researchers who have found reports on nurses who felt isolated and lacking adequate support from existing systems, respectively (Putri et al., 2021).

We also identified high rates of bullying experienced by nurses; this phenomenon includes various forms of verbal harassment, intimidation, and social exclusion in the workplace (Dousin et al., 2021; Goudarzian et al., 2024). Nurses who experienced workplace bullying were three times more likely to experience mental health disorders than nurses who did not experience bullying, with impacts that could persist for years after the incident and potentially affect the quality of care provided to patients (Hassanie et al., 2022; Kirkbride et al., 2024).

The coping ability of participants varied; some nurses reported that their coping was not good, while some felt resigned to returning to Indonesia and to the situation. Participants had to be able to manage themselves and be mentally always ready to learn, relieve stress with entertainment, find a place to express feelings, and strengthen commitment and planning. Researchers have shown significant variation in coping abilities among Indonesian nurses returning from work experiences abroad (Raharto and Noveria, 2023; Tsujita and Oda, 2023).

Some nurses still face various challenges and difficulties in their adaptation process, while other groups have succeeded in reaching the stage of acceptance and have resigned to their new situation in their homeland (Tsujita and Oda, 2023). The other groups of nurses who have succeeded at adaptation have implemented various coping strategies (Hassanie et al., 2022), such as: building mental readiness to continue learning and developing oneself continuously, implementing effective stress management techniques through various meaningful recreational activities, actively seeking and building social support networks as a place to express feelings and share experiences, and developing a strong professional commitment, accompanied by clear and structured career planning (Raharto and Noveria, 2020; Jang, Son and Lee, 2022). Researchers have identified that the level of nurses' resilience was greatly influenced by their ability to manage expectations realistically and their adaptive abilities in adjusting to the dynamics and demands of the new work environment in Indonesia (Kirkbride et al., 2024). We have emphasised the importance of developing holistic and sustainable adaptation strategies.

## 5 Strengths and limitations

This study considered several methodological aspects to ensure credibility. To enhance transparency, we described all stages of data collection and analysis, including clear profiles of informants, which also supports transferability. Contextual factors in the lives of Indonesian immigrant nurses were emphasised, particularly their mental health, with most informants holding higher education, a factor linked to successful personal, social, and economic adaptation. Credibility was also strengthened by recording and transcribing interviews to ensure data quality. The study, however, has limitations. Transferability is restricted to Indonesian nurses working abroad and may not apply to other immigrant groups. Future studies should examine nurses from different countries with similar experiences. Our focus on stress and pressure in a cross-national context may not fully capture the complexity of lived experiences. Time and accessibility constraints limited the number of in-depth interviews, and the short research period prevented analysis of long-term changes in stress and coping strategies. These limitations should be taken into account in future research, which could explore similar topics using broader samples, longer study periods, or alternative methodological approaches.

## 6 Conclusion

Indonesian nurses working abroad face a number of complex challenges as migrant nurses. Many participants reported significant mental health dynamics, including ongoing stress, deep mental distress, persistent fear, difficulty in dealing with substantial cultural differences, and severe challenges in adapting to a new environment. Participants reported the protection from Indonesian associations is needed for those working abroad. More feasibility studies may be required on rules and policies related to protection, in collaboration with government agencies, healthcare institutions, and nursing support programs.

## Funding

This research was part of a collaborative research project with Airlangga University and Andalas University, which has received funding from the Directorate General of Higher Education through the Catalyst Grant Scheme on the BIMA page. Based on Decree Number 062/E5/PG.02.00/PL.BATCH.2/2024 and Contract Agreement Number 004/SP2H/PT-BATCH.2/LL7/2024. The Directorate General of Higher Education was not involved in the research design, data collection, analysis, interpretation, or writing of the manuscript.

## Availability of data and materials

The datasets generated during the current study are not publicly available due to the nature of the e-supervisor and personal information contained in the data. Data may be available from the current author, with their supervisor and after ethical approval.

## CRediT authorship contribution statement

**Mundakir Mundakir:** Supervision, Methodology, Formal analysis, Data curation, Conceptualization. **Ferry Efendi:** Writing – review & editing, Supervision, Investigation. **Hema Malini:** Writing – review & editing, Supervision, Software. **Reliani Reliani:** Writing – original draft, Methodology, Data curation, Conceptualization. **Diah Priyantini:** Writing – original draft, Methodology, Formal analysis, Data curation, Conceptualization. **Chlara Yunita Prabawati:** Writing – original draft, Methodology, Formal analysis, Data curation, Conceptualization. **Rifky Octavia Pradipta:** Writing – original draft.

## Declaration of competing interest

The authors declare that they have no known competing financial interests or personal relationships that could have appeared to influence the work reported in this paper.
